# Perspectives of staff nurses of the reasons for and the nature of patient-initiated call lights: an exploratory survey study in four USA hospitals

**DOI:** 10.1186/1472-6963-10-52

**Published:** 2010-02-26

**Authors:** Huey-Ming Tzeng

**Affiliations:** 1 The University of Michigan, School of Nursing, Division of Nursing Business and Health Systems, 400 North Ingalls, Room 4156, Ann Arbor, MI 48109, USA

## Abstract

**Background:**

Little research has been done on patient call light use and staff response time, which were found to be associated with inpatient falls and satisfaction. Nurses' perspectives may moderate or mediate the aforementioned relationships. This exploratory study intended to understand staff's perspectives about call lights, staff responsiveness, and the reasons for and the nature of call light use. It also explored differences among hospitals and identified significant predictors of the nature of call light use.

**Methods:**

This cross-sectional, multihospital survey study was conducted from September 2008 to January 2009 in four hospitals located in the Midwestern region of the United States. A brief survey was used. All 2309 licensed and unlicensed nursing staff members who provide direct patient care in 27 adult care units were invited to participate. A total of 808 completed surveys were retrieved for an overall response rate of 35%. The SPSS 16.0 Window version was used. Descriptive and binary logistic regression analyses were conducted.

**Results:**

The primary reasons for patient-initiated calls were for toileting assistance, pain medication, and intravenous problems. Toileting assistance was the leading reason. Each staff responded to 6 to 7 calls per hour and a call was answered within 4 minutes (estimated). 49% of staff perceived that patient-initiated calls mattered to patient safety. 77% agreed that that these calls were meaningful. 52% thought that these calls required the attention of nursing staff. 53% thought that answering calls prevented them from doing the critical aspects of their role. Staff's perceptions about the nature of calls varied across hospitals. Junior staff tended to overlook the importance of answering calls. A nurse participant tended to perceive calls as more likely requiring nursing staff's attention than a nurse aide participant.

**Conclusions:**

If answering calls was a high priority among nursing tasks, staff would perceive calls as being important, requiring nursing staff's attention, and being meaningful. Therefore, answering calls should not be perceived as preventing staff from doing the critical aspects of their role. Additional efforts are necessary to reach the ideal or even a reasonable level of patient safety-first practice in current hospital environments.

## Background

Patient call light usage and nurse responsiveness to call lights are two intertwined concepts that could pertain to patients' safety during hospital stays [1,2]. However, little research has been done on this topic. In general, patient- or family-initiated call light usage is associated with how often patients or family visitors have unmet needs and require assistance. Response time to call lights is primarily determined by the nurses' reaction to each call light and may be linked to the circumstances present when a call is initiated.

Note that the present system of hospital care is based on the assumption that patients are able to get help by activating their nurse call system (also called call light or call bell). However, cognitive impairment, visual loss, and decreased mobility could make it difficult for patients to use the nurse call system when they need help. Inability to call for help (e.g., the call light panel or button is not within reach) may result in hospital-acquired harm (e.g., falling out of the bed due to un-assisted transfer) [3]. A study [4] identified multiple extrinsic risk factors for falls related to human involvement including: difficulties in determining patient care priorities, nursing staff's misconception about the purpose of call lights, call lights not being answered in a timely manner, difficulty in implementing timed observation and toileting plans, and patient assignments not being in close proximity, which may delay the responses to patients' call lights and needs. In a study related to fall prevention efforts [5], recently discharged older patients emphasized that nurses should provide assistance and answer a call light in a timely manner and a major safety concern during hospital stays was lack of availability of nurses to help when needed.

In addition, two studies [1,2] found that more calls for assistance were related to less fall-related patient injuries per 1000 patient-days and longer call light response times. When the average response time to call lights was longer, the patient satisfaction scores were lower [1]. A recent study [6] also examined the correlation between the patient satisfaction at discharge in relation to the number of the call light requests from the patient's room and staff response time in a 32-bed surgical unit. However, no statistically significant relationships were found. It's arguable that nurses' perspectives about patient-initiated call lights may moderate or mediate the relationships among patient call light usage, nurse call light responsiveness, and patient satisfaction with nursing care.

### The nurse call system and issues relevant to nursing practice

The call light is a vital patient communication link during hospital stays and is actually one of the few means by which cognitively intact patients can exercise some meaningful control over their care. The commonly adopted nurse call system features have the ability of allowing unit clerks to receive and screen patient calls in the nurses' station, which is meant to reduce unnecessary nurse interruptions. Such features may save actual nursing time, and enable some nurses to begin preparing to meet patients' needs before entering their rooms. Problems with the commonly adopted nurse call system are, for example: inability to locate the nurse, inability to prioritize and confirm calls, and inability to speak directly to patients and staff [7].

Call light technology has continued to develop (e.g., Vocera integration with a nurse call system). Enhanced nurse call systems have sought to provide more than a means for beckoning nursing personnel to the patient's room and to significantly increase their functionality. Although these advances provide improvements for workflow and offer an opportunity to improve response times, none of these systems have been shown to improve efficiencies, patient safety or reduce costs [8-10].

Hospitalized patients spend most of their time in their room necessitating use of call lights to have their needs met. Previous studies [11-14] have identified possible reasons why patients and families use call lights, including (but not limited to): (1) urgent calls, (2) toileting assistance, (3) intravenous problems, (4) pain medication, (5) repositioning and transfer assistance, (6) personal assistance (e.g., for food, water), (7) obtaining information, (8) getting nurses' attention, (9) asking for nursing staff's companionship, and (10) accidentally pushing the call light.

It is commonly assumed that if a nurse responds to a call light more quickly, the patient may have less opportunity to fall. However, call lights are perceived by some nurses as mere noise and an interruption to nursing tasks, instead of an important way for patients to request assistance [12]. Deitrick and associates [11] examined problems related to answering patient call lights in acute inpatient care settings and found that the most frequent comments of patients were: (1) delays in getting call lights answered, (2) variation in the call light response time from a low of less than a minute to a high of 20 minutes, (3) the amount of time it took to handle the patient's request once the light was answered, and (4) the patient's request not being fulfilled once the call light was answered. Frustration over delays in answering call lights is one of the most frequent comments that patients make.

In short, little research has been done on patient call light use and staff call light response time, and only one small-scale pilot study [15] intended to understand how nursing staff view patient-initiated call lights. This pilot study [15] found that, although the majority of the staff (81.6%) agreed that call lights were meaningful, only half of the staff members perceived that call lights mattered to patient safety and required nursing staff attention and 44% thought that answering call lights prevented them from doing critical aspects of their role.

### Theoretical framework

The National Quality Forum (NQF) [16] suggested outcome, process, structure, and patient-centered measures as considerations for supporting internal healthcare organization quality improvement. Using NQF's approach for falls' assessment, the outcome measures include: (1) falls with injuries, and (2) falls prevalence. The process measures intend to quantify the level of staff adherence to organizational policy that represents effective falls prevention practices, including: (1) percentage of patients screened for falls, and (2) percentage of patients educated about fall prevention strategies and risks. The structure measures include: (1) the presence of an organizational falls prevention policy, and (2) the presence of measurable structures in place to ensure accountability for performance. The patient-centered measures refer to evidence that patients' values and preferences are respected.

Previous studies [4,16-18] suggested that intrinsic (those integral to the patient) and extrinsic (those external to the patient) risk factors for fall and injurious fall occurrence can be interwoven with each other causing an even greater risk together than separately. The intrinsic risk factors are composed of, but not limited to, patients' demographics, cognitive functioning, functioning status, physiologic status, primary patient medical condition, acuity level, length of stay, and *call light usage*. The extrinsic risk factors include, but are not limited to, risks related to environment (e.g., design of furnishings and equipments, patient-staff communication devices), treatment and medications, staffing, use of unlicensed personnel (e.g., sitters, family visitors), and nursing actions to address patient needs (e.g., *staff response time to patient needs*, fall prevention protocol).

**Figure 1:**
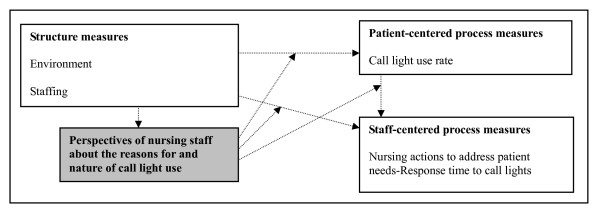
**The conceptual model of this study**. This study explored the perspectives of nursing staff about the reasons for and the nature of call light use; it did not test the relationships between the variables in the boxes.

Figure 1 was developed to illustrate the significance of this study within the context of patient safety with a focus on inpatient falls. This figure used a macro-view to depict the importance of this subject. Staff's response time to call lights was categorized as a staff-centered process measure and patient call light use rate was a patient-centered process factor. It was assumed that nurses' perspectives about call lights may moderate or mediate the relationships among patient call light usage, nurse responsiveness, and patient satisfaction. Consequently, this project was proposed to explore nurses' viewpoints.

### Purpose of this study and research questions

This exploratory, cross-sectional, multihospital survey study attempted to understand staff's perspectives about patient-initiated call lights, staff responsiveness, and the reasons for and nature of call light use. It also explored the differences in the perspectives of staff members in four hospitals. In addition, it was designed to identify significant predictors of the nature of call light use. As an exploratory study, the candidate predictors included: hospital, staff's age, tenure, gender, job title, educational background, unit type, and primary working shift. These were limited to the indicators collected in the survey as a study limitation. To correspond to the study purposes, this study answered five research questions:

(1) What were the reasons for call lights as perceived by staff members?

(2) How many call lights per hour did a staff member respond to alone?

(3) How long did it take to answer a call light during the day, evening, and night shifts?

(4) How did a staff member perceive the nature of call lights?

(5) What were the significant predictors for staff's perspectives about the nature of call lights?

## Methods

### Design

This exploratory, cross-sectional, multihospital survey study was conducted from September 2008 to January 2009 at four hospitals located in the Midwestern region of the United States. The study was approved by each hospital's institutional review board and the author's employer university (HUM22711). There was no conflict of interest.

### Sample and setting

A convenient sample was used, due to limited budget, and only four acute hospitals were recruited to participate in this study, including a total of 27 adult inpatient acute care units. The numbers of study units and bed size for each respective study hospital were: Hospital 1, an academic medical center (12 units, bed size about 900); Hospital 2, a community teaching hospital (4 units, bed size about 300); Hospital 3, a veteran affairs medical center (3 units, bed size about 100); and Hospital 4, a teaching medical center (8 units, bed size about 700). All these 27 study units have a nurse call system in place. In order to maximize the number of the completed surveys, all 2309 licensed and unlicensed nursing staff members who provide direct patient care in the study adult care units, as the targeted population, were invited to participate.

### Instrumentation and data collection

The call light staff survey questionnaire (16 items of the nurse self-reported measures) used in this paper has been pilot tested in a government hospital [15] (Table 1 and Additional file 1). These items were composed based on the previous studies [11-14] and developed for the purpose of this multihospital study. This survey has content validity; nine clinical experts reviewed each item for appropriateness and word use. Unfortunately, content validity indexes on the experts' comments were not performed. As a result of the experts' comments, only the use of words in the survey was modified for increased clarity. This survey was previously pre-tested for clarity and time/ease of completion by 38 staff nurses and nurse aides, who worked in an acute medical-surgical unit at a Michigan academic medical center. No changes were made based on the outcomes of the pre-test and pilot test. Because all the questions are single-item scales, a reliability analysis was not conducted [15].

**Table 1:** Items included in the call light staff survey questionnaire

Demographic characteristics
1. Gender (1 = Male, 0 = Female)
2. Age in years
3. Highest completed education program (1 = High school or diploma, 2 = Associate degree, 3 = Bachelor's degree, 4 = Master's degree or higher)
4. Type of unit (the unit the participant was working for, as identified by the participant) (1 = Acute medical, 2 = Acute surgical, 3 = Acute medical-surgical combined, 4 = Birthing center, 5 = Step-down medical, 6 = Step-down medical-surgical combined, 7 = Floating)
5. Work title (1 = Staff nurse, including registered nurse and licensed practical nurse, 2 = Nurse aide, nurse technician, or medical assistant, 3 = Unit clerk or administrative assistant, 4 = Psych technician)
6. Primary working shift (1 = Day shift, 2 = Evening shift, 3 = Night shift, 4 = 12-hour day shift, 5 = 12-hour night shift, 6 = Rotating)
7. Years working in acute inpatient care units (in years)

**Call light related items**

8. Selection of the reasons for patient-initiated call lights and the prevalence of each reason, by percentage. The reasons include:
(1) Extremely urgent medical problem (e.g., like a 911 life-or-death emergency call in the United States) (1 = Yes)
(2) Bathroom, bedside commode, or bedpan assistance (1 = Yes)
(3) Intravenous problems or pump alarm (1 = Yes)
(4) Pain medication and management (1 = Yes)
(5) Repositioning, transfer, or mobility assistance (1 = Yes)
(6) Personal assistance (1 = Yes)
(7) Obtaining information (1 = Yes)
(8) Getting nurses' attention for no specific reason (1 = Yes)
(9) Demanding a nurse's presence or a companion at bedside for no specific reason (1 = Yes)
(10) Accidentally pushed the call light (1 = Yes)
9. Number of patient-initiated calls per hour the individual responds to (number of calls, estimated)
10. Number of patient-initiated calls per hour the individual and team members together respond to (e.g., nurse, nurse aide, nurse technician, or medical assistant) (number of calls, estimated)
11. Average length of time to answer a patient-initiated call light during the day, evening, and night shift, respectively (number of minutes, specified by shift, estimated)
12. Opinion whether most of the call lights pertain to patients' safety during hospital stays? (1 = Yes, 0 = No)
13. Opinion whether most of the call lights require nursing staff's attention and nursing care? (1 = Yes, 0 = No)
14. Opinion whether most of the reasons for call lights are meaningful? (1 = Yes, 0 = No)
15. Opinion whether answering call lights prevents you from doing the critical aspects of your role? (1 = Yes, 0 = No) If yes, why? (an open question, a qualitative variable)
16. Solicitation of opinions identifying matters or issues that have a higher priority than answering patient-initiated call lights (an open question; a qualitative variable)

The items used to measure the studied concepts included: staff's perspectives about patient-initiated call lights (Item 9), staff responsiveness (Item 11), the reasons for call light use (Item 8), and the nature of call light use (Items 12-15). The candidate predictors of the nature of call light use included hospital (documented by the author), staff's age (Item 2), tenure (Item 7), gender (Item 1), job title (Item 5), educational background (Item 3), unit type (Item 4), and primary working shift (Item 6). Specifically, for the responses collected within Item 8 (the reasons for call light use), the indicated prevalence (in percentage) was then ranked among the identified situations within each completed survey and recoded for further data analysis (1 = The most prevalent reason). Each participant may assign two or more than two of the selected reasons the same frequency/prevalence level. As a result, two or more than two of the selected reasons may be ranked as the most prevalent reason within a completed survey.

The survey packages were left in each potential participant's mailbox located in the staff lounge. Staff participants received a copy of the survey questionnaire and an information sheet about this study. Participation was voluntary and anonymous, with no identifiers recorded or tracked on the survey questionnaire. Return of the completed surveys indicated consent. To prompt completion, a reminder follow-up letter was sent to all possible participants two weeks after the survey was disseminated.

### Data analyses

The Statistical Package for the Social Sciences (16.0 Window version; SPSS Inc., Chicago, IL, USA) was used for data analyses. Descriptive analyses were used to answer the first four research questions. To answer the fifth question, four binary logistic regression models were developed to determine significant predictors (alpha was set at .05) of four dependent variables respectively, including: (1) important to patients' safety, (2) requiring nursing staff's attention, (3) reasons for call lights being meaningful, and (4) action of answering call lights preventing staff from doing critical aspects of their role. Candidate predictors (hospital, staff member's age, tenure, gender, job title, educational background, unit type, and primary working shift) were entered into the models at the same time. Dummy variables were created for the categorical variables (hospital, gender, job title, educational background, unit type, and primary working shift); the number of created dummy variables for each categorical variable was: the number of categories minus 1. It resulted in a total of 17 predicators in each regression model. The responses of all participants (nursing staff and nurse aides) were included in the analyses.

## Results

A total of 808 completed surveys were retrieved from a total of 27 adult inpatient acute care units at four hospitals for an overall response rate of 35%. The numbers of valid surveys for each respective study hospital were: Hospital 1, an academic medical center (n = 459); Hospital 2, a community teaching hospital (n = 119); Hospital 3, a veteran affairs medical center (n = 47); and Hospital 4, a teaching medical center (n = 183). Among these participants, 636 (79%) staff nurses (registered nurses and licensed practical nurses) and 172 (21%) nurse aides returned the completed surveys study.

For the entire group of participants, Pearson *χ*^2 ^tests showed significant associations between hospitals and the proportions of participants' highest completed education level, work title, unit type, and primary working shift. One-way ANOVA tests found significant differences in the participants' mean age and tenure in years across four study hospitals; the staff participants from Hospital 3 had the highest mean in age (mean = 44.57 years) and those from Hospital 2 had the longest mean in tenure (mean = 9.94 years) (Table 2). The following sub-sections presented the answers to the specific research questions by the theme of the research question.

**Table 2:** Characteristics of the study sample

	All (n = 808)	Hosp. 1 (n = 459)	Hosp. 2 (n = 119)	Hosp. 3 (n = 47)	Hosp. 4 (n = 183)	
**Variable/Frequency (valid percent)**	**Freq. (%)**	**Freq. (%)**	**Freq. (%)**	**Freq. (%)**	**Freq. (%)**	**Pearson χ^2 ^test (*P*)**

Gender						*χ*^2 ^= 4.78 (*P *= .19)
Male	73 (9)	46 (10)	7 (6)	7 (15)	13 (7)	
Female	735 (91)	413(90)	112 (94)	40 (85)	170 (93)	
Highest completed education program						*χ*^2 ^= 38.85 (*P *= .001**, Phi = .23)
High school or diploma	81 (11)	38 (9)	23 (22)	4 (9)	16 (9)	
Associate degree	337 (44)	176 (40)	58 (55)	16 (38)	87 (51)	
Bachelor's degree	316 (41)	208 (47)	22 (21)	20 (48)	66 (38)	
Master's degree or higher	28 (4)	20 (4)	2 (2)	2 (5)	4 (2)	
Work title						*χ*^2 ^23.82 (*P *= .001**, Phi = .17)
Staff nurse	636 (79)	374 (82)	74 (62)	41 (87)	147 (80)	
Nurse aide/technician	172 (21)	85 (18)	45 (38)	6 (13)	36 (20)	
Type of unit						*χ*^2 ^= 148.28 (*P *= .001**, Phi = .43)
Acute medical	406 (50)	279 (61)	51 (43)	38 (81)	38 (21)	
Acute surgical	128 (16)	27 (6)	30 (25)	9 (19)	62 (34)	
Acute medical-surgical	274 (34)	153 (33)	38 (32)	-	83 (45)	
Primary working shift						*χ*^2 ^= 134.90 (*P *= .001**, Phi = .41)
Day shift	226 (33)	139 (30)	38 (33)	23 (49)	66 (37)	
Evening shift	70 (9)	54 (12)	6 (5)	8 (17)	2 (1)	
Night shift	76 (9)	54 (12)	9 (8)	3 (7)	10 (6)	
12-h day shift	149 (19)	49 (10)	43 (37)	2 (4)	55 (30)	
12-h night shift	150 (19)	81 (18)	15 (13)	10 (21)	44 (24)	
Rotating	90 (11)	81 (18)	4 (4)	1 (2)	4 (2)	

**Variable/Mean (standard deviation)**	**Mean (SD)**	**Mean (SD)**	**Mean (SD)**	**Mean (SD)**	**Mean (SD)**	**One-way ANOVA (*P*)**

Age	36.67 (11.19)	35.92 (10.82)	38.36 (12.07)	**44.57 **(11.92)	35.31 (10.41)	F = 10.31, (*P *= .001**)
Tenure in acute inpatient care units	7.32 (8.30)	6.47 (7.74)	**9.94 **(9.87)	9.91 (8.63)	7.07 (8.05)	F = 6.94 (*P *= .001**)

**P < .01.

### Reasons for call light use

Based on all the respondents, among the 10 mentioned possibilities, the reasons identified by at least 90% of the participants for call light use were: (1) pain medication and management (the most often identified reason), (2) bathroom assistance, (3) intravenous problems or pump alarm, (4) personal assistance, (5) accidental pressing of the call light, and (6) repositioning or transfer (Table 3).

**Table 3:** Call-light related characteristics: The results as the variables reported by frequencies

	All (n = 808)	Hosp. 1 (n = 459)	Hosp. 2 (n = 119)	Hosp. 3 (n = 47)	Hosp. 4 (n = 183)	
**Variable/Frequency (valid percent)**	**Freq. (%)**	**Freq. (%)**	**Freq. (%)**	**Freq. (%)**	**Freq. (%)**	

Selection of the reasons of patient-initiated call lights:						Note: For each reason, the frequencies that were ≥ 90% are in bold.
(1) Extremely urgent medical problem	546 (68)	318 (69)	78 (66)	31 (66)	119 (65)	
(2) Bathroom assistance	**778 (96)**	**442 (96)**	**114 (96)**	**45 (96)**	**177 (97)**	
(3) Intravenous problems/pump alarm	**770 (95)**	**436 (95)**	**114 (96)**	**46 (98)**	**174 (95)**	
(4) Pain medication and management	**779 (96)**	**442 (96)**	**114 (96)**	**47 (100)**	**176 (96)**	
(5) Repositioning or transfer	**746 (92)**	**425 (93)**	**110 (92)**	**43 (92)**	**168 (92)**	
(6) Personal assistance	**758 (94)**	**428 (93)**	**111 (93)**	**46 (98)**	**173 (95)**	
(7) Obtaining information	680 (84)	375 (82)	105 (88)	**43 (92)**	157 (86)	
(8) Getting attention only	609 (75)	357 (78)	86 (72)	39 (83)	127 (69)	
(9) Demanding a nurse's presence	655 (81)	371 (81)	101 (85)	39 (83)	144 (79)	
(10) Accidentally pushed the call light	**749 (93)**	**425 (93)**	**108 (91)**	**43 (92)**	**173 (95)**	

The frequency of each identified reason being ranked as the most prevalent reason:						Note: Each participant may assign two or more than two of the selected reasons the same frequency level. The highest three frequencies for each reason are in bold.
(1) Extremely urgent medical problem	7 (1.5)	3 (1)	2 (3)	2 (7)	1 (1)	
(2) Bathroom assistance	**438 (57)**	**217 (50)**	**69 (62)**	**26 (58)**	**126 (72)**	
(3) Intravenous problems/pump alarm	**193 (25)**	**127 (29)**	**23 (21)**	**12 (26)**	31 (18)	
(4) Pain medication and management	**324 (42)**	**196 (45)**	**36 (32)**	**17 (36)**	**75 (43)**	
(5) Repositioning or transfer	78 (11)	35 (8)	6 (6)	6 (14)	31 (19)	
(6) Personal assistance	130 (17)	72 (17)	16(15)	9 (20)	**33 (19)**	
(7) Obtaining information	28 (4)	7 (2)	5 (5)	6 (14)	10 (7)	
(8) Getting attention only	23 (4)	12 (4)	4 (5)	4 (11)	3 (3)	
(9) Demanding a nurse's presence	19 (3)	11 (3)	2 (2)	1 (10)	2 (2)	
(10) Accidentally pushed the call light	20 (3)	11 (3)	2 (2)	6 (14)	1 (1)	

**Variable/Frequency (valid percent)**	**Freq. (%)**	**Freq. (%)**	**Freq. (%)**	**Freq. (%)**	**Freq. (%)**	**Pearson *χ*^2 ^**test (*P*)

Opinion whether most of the call lights matter to patients' safety?						*χ*^2 ^= 31. 78 (*P *= .001**, Phi = .21)
Yes	373 (49)	182 (42)	50 (49)	**24 (55)**	**117 (67)**	
No	**380 (51)**	**225 (58)**	**53 (51)**	22 (46)	57 (33)	
Opinion whether most of the calls require nursing staff's attention and nursing care?						*χ*^2 ^= 4.02 (*P *= .26)
Yes	**406 (52)**	**227 (51)**	**68 (60)**	**25 (54)**	86 (49)	
No	372 (48)	215 (49)	45 (40)	21 (46)	**91 (51)**	
Opinion whether most of the reasons for call lights are meaningful?						*χ*^2 ^= 7.16 (*P *= .07)
Yes	**604 (77)**	**328 (74)**	**89 (79)**	**39 (83)**	**148 (83)**	
No	177 (23)	115 (25)	24 (21)	8 (17)	30 (17)	
Opinion whether answering call lights prevents you from doing the critical aspects of your role?						*χ*^2 ^= 9.25 (*P *= .03*, Phi = .11)
Yes	**408 (53)**	**250 (57)**	**61 (56)**	21 (46)	75 (44)	
No	356 (47)	189 (43)	48 (44)	**25 (54)**	**94 (56)**	

*P < .05.

**P < .01.

Based on the frequency of each identified reason being ranked as the most prevalent reason within each respondent, the reason for bathroom assistance was most often identified as the leading reason for call light use, followed by need for pain medication and management, and intravenous problems or pump alarm. In other words, staff members most often identified bathroom assistance as the most common reason for call light use (Table 3).

### Number of call light responded by staff

Based on staff's recall, on average, each staff member responded to 6.46 calls per hour, ranging from 4.80 calls (Hospital 3) to 6.83 calls (Hospital 1). In other words, each staff may respond to about 52 calls during an 8-hour shift or 78 calls during a 12-hour shift (about one call every 9 minutes). No significant differences were found across the four hospitals in the means of the number of calls per hour to which an individual staff person responded (Table 4).

**Table 4:** Call-light related characteristics: The results as the variables reported by means

	All (n = 808)	Hosp. 1 (n = 459)	Hosp. 2 (n = 119)	Hosp. 3 (n = 47)	Hosp. 4 (n = 183)	
**Variable/Mean (Standard deviation)**	**Mean (SD)**	**Mean (SD)**	**Mean (SD)**	**Mean (SD)**	**Mean (SD)**	**One-way ANOVA tests (*P*)**

No. of calls per hour a staff responds to	6.46 (6.99)	6.83 (8.23)	6.32 (5.04)	4.80 (3.31)	6.05 (4.98)	F = 1.47, (*P *= .22)
Average length of time to answer a call light during day shift, min	3.57 (2.56)	3.63 (2.51)	3.47 (2.13)	2.42 (1.55)	3.78 (3.04)	F = 2.34, (*P *= .07)
Average length of time to answer a call light during evening shift, min	3.70 (2.72)	3.68 (2.80)	3.73 (2.67)	2.62 (1.79)	4.05 (2.63)	F = 1.50, (*P *= .21)
Average length of time to answer a call light during night shift, min	3.42 (3.38)	3.49 (3.36)	2.78 (2.08)	1.99 (1.48)	3.38 (4.06)	F = 2.09, (*P *= .10)

### Response time to call lights

The average length of time to answer a call light was 3.57 minutes during day shifts, 3.70 minutes during evening shifts, and 3.42 minutes during night shifts. Overall, regardless of different shifts, a call was expected to be answered with 4 minutes or 3 minutes and 42 seconds. No significant differences were noted across the four hospitals in the means of the self-reported response time to call lights (Table 4).

### Nature of call lights

A little less than half of the participants (n = 373, 49%) perceived that call lights mattered to patient safety, 406 (52%) thought call lights required nursing staff attention, 604 (77%) considered them meaningful, and 480 (53%) thought that answering call lights prevented them from doing critical aspects of their role. Pearson χ^2 ^tests showed significant differences between hospitals on staff's perceptions about call light importance to patients' safety and on staff's perception that answering call lights prevented them from doing critical aspects of their role (Table 3).

### Predicting the perceived nature of call lights

The results of four logistic regression models were presented in Table 5. The perceptions of all participants (nursing staff and nurse aides) were included in the analyses. The dependent variables for these four regression models were: (1) important to patients' safety, (2) requiring nursing staff's attention, (3) reasons for call lights being meaningful, and (4) action of answering call lights preventing staff from doing critical aspects of their role. Candidate predictors (hospital, staff member's age, tenure, gender, job title, educational background, unit type, and primary working shift) were entered into the models at the same time. Dummy variables were created for the categorical variables (hospital, gender, job title, educational background, unit type, and primary working shift). A total of 17 candidate predicators were entered into each regression model.

**Table 5:** Summary of the results of the binary logistic regression analyses

Dependent Variable/Independent Variable	Cox & Snell R^2^	Nagelkerke R^2^	Percentage correct^a^
**Model 1: **Opinion whether most of the call lights matter to patients' safety? (1 = yes, 0 = no)	.06	.08	59.7%
**Model 2: **Opinion whether most of the call lights require nursing staff's attention and nursing care? (1 = yes, 0 = no)	.11	.15	63.5%
**Model 3: **Opinion whether most of the reasons for call lights are meaningful? (1 = yes, 0 = no)	.06	.10	78%
**Model 4: **Opinion whether answering call lights prevents you from doing the critical aspects of your role? (1 = yes, 0 = no)	.07	.09	59.1%

**Model 1: **Opinion whether most of the call lights matter to patients' safety? (1 = yes, 0 = no)	**B**	**Wald**	**Sig**.

Hospital (overall)	-	**23.94**	.001**
Hospital 1	**-1.04**	**19.72**	.001**
Hospital 2	**-.84**	**7.56**	.006**
Hospital 3	**-**.13	.11	.74
Age in years	**-**.01	.11	.74
Tenure in years	.01	1.03	.31
Gender (1 = male)	.09	.09	.76
Job title (1 = nurse)	.48	2.38	.12
Education (overall)	-	.95	.81
Education: High school or diploma	.20	.10	.75
Education: Associate degree	.33	.43	.51
Education: Bachelor's degree	.41	.68	.41
Unit type (overall)	-	.49	.78
Unit type: Acute medical unit	**-**.13	.49	.49
Unit type: Acute surgical unit	**-**.06	.05	.83
Shift (overall)	-	4.80	.44
Shift: Day shift	**-**.19	.42	.52
Shift: Evening shift	**-**.58	2.42	.12
Shift: Night shift	.20	.29	.59
Shift: 12-hour day shift	**-**.06	.03	.86
Shift: 12-hour night shift	**-**.06	.04	.84
Constant	.44	.36	.55

**Model 2: **Opinion whether most of the call lights require nursing staff's attention and nursing care? (1 = yes, 0 = no)	**B**	**Wald**	**Sig**.

Hospital (overall)	-	1.18	.75
Hospital 1	**-**.01	.001	.98
Hospital 2	.25	.68	.41
Hospital 3	.22	.30	.59
Age in years	**-**.02	2.01	.16
Tenure in years	.03	3.19	.07
Gender (1 = male)	.46	2.38	.12
Job title (1 = nurse)	**1.76**	**23.83**	.001**
Education (overall)	-	2.64	.45
Education: High school or diploma	**-**.40	.41	.52
Education: Associate degree	**-**.35	.56	.45
Education: Bachelor's degree	**-**.08	.03	.87
Unit type (overall)		3.93	.14
Unit type: Acute medical unit	.35	3.17	.08
Unit type: Acute surgical unit	.38	2.16	.14
Shift (overall)	-	**12.82**	.03*
Shift: Day shift	**-**.47	2.52	.11
Shift: Evening shift	**-**.25	.45	.50
Shift: Night shift	.62	2.67	.10
Shift: 12-hour day shift	**-**.27	.63	.43
Shift: 12-hour night shift	**-**.42	1.74	.19
Constant	**-**.05	.01	.94

**Model 3: **Opinion whether most of the reasons for call lights are meaningful? (1 = yes, 0 = no)	**B**	**Wald**	**Sig**.

Hospital (overall)	-	**8.99**	.03*
Hospital 1	**-.78**	**7.31**	.007**
Hospital 2	**-.77**	**4.49**	.03*
Hospital 3	**-**.20	.15	.70
Age in years	**-**.01	1.01	.32
Tenure in years	**.07**	**12.26**	.001**
Gender (1 = male)	**-**.18	.26	.61
Job title (1 = nurse)	.67	2.83	.09
Education (overall)	-	1.37	.71
Education: High school or diploma	**-**.56	.54	.46
Education: Associate degree	**-**.12	.04	.84
Education: Bachelor's degree	.01	.01	.99
Unit type (overall)	-	.53	.77
Unit type: Acute medical unit	**-**.12	.29	.59
Unit type: Acute surgical unit	**-**.20	.43	.51
Shift (overall)	-	**17.99**	.003**
Shift: Day shift	**-1.09**	**8.14**	.004**
Shift: Evening shift	.58	.99	.32
Shift: Night shift	**-**.72	2.54	.11
Shift: 12-hour day shift	**-**.83	3.67	.06
Shift: 12-hour night shift	**-1.08**	**7.29**	.007**
Constant	2.90	10.43	.001**

**Model 4: **Opinion whether answering call lights prevents you from doing the critical aspects of your role? (1 = yes, 0 = no)	**B**	**Wald**	**Sig**.

Hospital (overall)	-	**14.74**	.002**
Hospital 1	**.86**	**13.61**	.001**
Hospital 2	**.62**	**4.40**	.04*
Hospital 3	.33	.67	.41
Age in years	.02	3.39	.07
Tenure in years	**-**.01	.43	.51
Gender (1 = male)	.10	.13	.72
Job title (1 = nurse)	**-**.53	3.09	.08
Education (overall)	--	1.96	.58
Education: High school or diploma	**-**.54	.85	.36
Education: Associate degree	**-**.16	.12	.73
Education: Bachelor's degree	**-**.34	.55	.46
Unit type (overall)	-	2.76	.25
Unit type: Acute medical unit	**-**.01	.01	.98
Unit type: Acute surgical unit	.40	2.36	.12
Shift (overall)	-	**12.02**	.04*
Shift: Day shift	.30	1.07	.30
Shift: Evening shift	**-**.24	.43	.51
Shift: Night shift	.04	.01	.91
Shift: 12-hour day shift	**.84**	**6.24**	.01*
Shift: 12-hour night shift	.39	1.55	.21
Constant	**-**1.26	3.15	.08

^a^Percentage correct refers to the overall percentage of correctly predicted values reported in the classification table. The cut-off value is .50.

*P < .05.

**P < .01.

The first binary logistic regression model with the dependent variable of *call lights being important to patients' safety *showed that only the categorical variable of hospital was a significant predictor, where a staff member, who worked in Hospital 1 or Hospital 2 would perceive call lights as being *less *important to patients' safety. In other words, only one significant predictor was identified (Table 5).

The second binary logistic regression model with the dependent variable of *call lights requiring nursing staff's attention *indicated that a nurse participant tended to perceive call lights as more likely to require nursing staff's attention than a nurse aide participant. In addition, the categorical variable of the respondent's primary working shift was a significant predictor; however, none of the shifts predicted the dependent variable (Table 5).

The third binary logistic regression model with the dependent variable of *the reasons for call lights being meaningful *showed that the categorical variable of the hospital was a significant predictor, where a staff member, who worked in Hospital 1 or Hospital 2, would perceive call lights as being less meaningful. If participants had longer tenure, they tended to perceive call lights as being more meaningful. The categorical variable of the respondent's primary working shift was a significant predictor, where a staff member, who worked in the day shift or 12-hour night shift, would perceive call lights as being less meaningful (Table 5).

The fourth binary logistic regression model with the dependent variable of *the action of answering call lights preventing the staff participants from doing critical aspects of their role *showed that the categorical variable of the hospital was a significant predictor, where staff members, who worked in Hospital 1 or Hospital 2, tended to perceive that the action of answering call lights would prevent them from doing critical aspects of their role. The categorical variable of the respondent's primary working shift was a significant predictor, where staff members, who worked on the 12-hour day shift, tended to perceive that the action of answering call lights would prevent them from doing critical aspects of their role (Table 5).

## Discussion

This exploratory, cross-sectional, multihospital survey study attempted to understand staff's perspectives about patient-initiated call lights, staff responsiveness, and the reasons for and the nature of call light use. It also determined the predictors of the nature of call light use, including these four dependent variables: (1) important to patients' safety, (2) requiring nursing staff's attention, (3) reasons for call lights being meaningful, and (4) action of answering call lights preventing staff from doing critical aspects of their role. The following sub-sections discussed the answers to the specific research questions by the theme of the research question.

### Reasons for call light use

The answer to *the first research question *suggested that the primary six reasons for patient-initiated call lights were: (1) pain medication and management, as the most often identified reason, (2) bathroom assistance, (3) intravenous problems or pump alarm, (4) personal assistance, (5) accidental pressing of the call light, and (6) repositioning or transfer. Toileting assistance was the leading reason for call light use. These findings suggested that if a hospital included rounding for patient comfort and safety [12,19] as one of the patient safety initiatives, such rounding should be oriented to patients' toileting needs, pain management, and intravenous problems (specified requests).

When targeting on a patient's specified requests, personal assistance (unspecified, orderly requests) may be addressed at the same time. The reason is that specified requests are more predictable and tend to require licensed nurses' attention than the unspecified ones, such as needs for water and reposition [20]. This study's findings also implied that licensed nurses should be the primary person responding to call lights.

In addition, the rounding schedule should be justified based on the frequency of each individual patient's needs and changes in medical conditions (e.g., post-operation). For example, an individual patient's toileting needs may vary over the entire course of hospitalization, due to changes in medication usage (e.g., diuretics, benzodiazepines, sedatives) and changes in dependency in transferring/mobility (e.g., postoperatively).

### Patient call light use and nurse responsiveness

The answers associated with *the second and third research questions *suggested that, on average, each staff member responded to 6 to 7 call lights per hour. The estimated length of time to answer a call light was within 4 minutes. It should be noted that the survey questionnaire did not ask participants to specify the number of calls they responded to and did not ask participants their response time to call lights based on the nature or types of call lights.

In most of the inpatient care settings, patient- or family-initiated call lights have been categorized into normal calls (made from the pillow speaker), urgent calls (when a normal call was not answered within 3 minutes, an urgent call will be sent out), or toileting or bathroom calls (the calls made from the bathroom). Few institutions have adopted newer pillow speaker technology, where patients can specify their needs by pushing the button for water, pain medication, or bathroom/bedpan assistance.

To endorse patient-centered care, nursing executives and unit managers must promote the effectiveness of patient-initiated call light use and the efficiency of staff's responsiveness to call lights. As a practical matter, upgrading the call light system technology is necessary to help nursing staff determine patient care priorities for the purpose of reducing patient injury and falls [20]. For example, the call light panel could have three options to indicate the urgency level of each patient- or family-initiated call: (1) urgent call (e.g., unexpected bleeding, shortness of breath, dizziness), (2) normal call (e.g., bathroom assistance, intravenous problems or pump alarm, pain medication and management), and (3) orderly assistance (e.g., repositioning, transfer or mobility assistance, personal assistance, obtaining information about medications and health status, demanding a nurse's companionship at bedside) [20].

### Nature of call lights

The answer to *the fourth research question *suggested that less than half (49%) of the participants perceived that patient-initiated call lights mattered to patient safety. Surprisingly, 77% of them agreed that that these calls were meaningful. In addition, only 52% thought that these calls required the attention of nursing staff. Consequently, it seems to be legitimate that up to 53% of the participants thought that answering call lights prevented them from doing the critical aspects of their role.

It was assumed that if answering call lights was prioritized higher among nursing tasks, a staff member would perceive call lights as being important to patient safety, requiring nursing staff's attention, and meaningful. If so, the action of answering call lights should not be perceived as preventing staff members from doing the critical aspects of their role.

Accordingly, it is suggested that regular on-the-job training of patient safety-first practices with a focus on addressing patients' call lights would be required to raise the consensus perception of the importance levels of each call light among staff members. Such educational interventions should also target improving the morale of staff members by acknowledging their efforts to promote patient safety. It is also essential to develop a simple, straightforward, feedback loop to staff on their performance from patients, families, and unit managers (e.g., quality of patient-nurse interaction, patients' need being addressed in a timely manner). Occasionally, incentives to staff would be needed to reinforce patient safety-first practices. Such incentives may be linked to the feedback mechanism.

### Predicting the perceived nature of call lights

The answers to *the fifth research question *suggested that if staff members worked in Hospital 1 or Hospital 2, they would tend to perceive call lights as being less important to patients' safety, as being less meaningful, and that the action of answering call lights would prevent them from doing critical aspects of the nursing role. In other words, staff's perceptions about the nature of call lights were found to vary significantly across hospitals. This difference can be due to the organization's patient safety culture or the leadership profiles of the hospital or nursing executives and middle-level and unit-level managers. However, this study did not measure the study hospitals' patient safety culture or leadership profiles, and is unable to test the aforementioned possible relationship.

Tenure was found to be a significant predictor of the reasons for call lights being meaningful. If a participant had longer tenure, he or she tended to perceived call lights as being more meaningful. This finding suggested that junior staff tended to overlook the importance of answering patient- or family-initiated call lights. Therefore, new staff orientation should include information related to patient safety-first practice with a focus on addressing patients' call lights.

In addition, a nurse participant tended to perceive call lights as more likely requiring nursing staff's attention than a nurse aide participant. Consequently, on-the-job training for patient safety-first practices should be tailored for nurse aides to be consistent with their roles in the process of delivering patient care. It may be appropriate to offer different patient safety-first practice on-the-job training sessions for nurses and nurse aides to address their specific requirements.

Also, staff members who worked a 12-hour day shift tended to perceive that the action of answering call lights would prevent them from performing critical aspects of their role. It is possible that the day shift has more procedures, treatments, admissions, or discharges that a nurse or nurse aide must handle than those working in evening and night shifts. The respondent's primary working shift was also a significant predictor of the reasons for call lights being meaningful. However, the predicting direction of the respondent's primary working shift was not conclusive, and further research is needed.

In short, the candidate predictors of hospital, tenure, job title, and primary working shift were found to affect at least one of the four dependent variables of the nature of call light use. The candidate predictors of staff member's age, gender, educational background, and unit type were not found to be significant predictors affecting any of the four dependent variables of the nature of call light usage.

These findings suggested that employment-related characteristics (hospital, tenure, job title, and primary working shift) were significant determinants of nurses' perceptions about the nature of call light use. The predictor of hospital could be a dominant characteristic that supersede the influence of unit type. The findings also suggested that individual staff's characteristics (age, gender, and educational background) should not be considered as candidate predictors in predicting nurses' perceptions on the issues related to the nature of call light use. The perceived nature of call light use could be a shared, hospital-wide phenomenon that may be linked to an organization's culture, instead of each individual staff's demographic characteristics.

### Study limitations and future research directions

The scope of this study is limited to four hospitals located in the Midwestern region of the United States, reducing the ability to generalize the findings. In addition, each hospital adopted similar but somewhat different call light-related devices or technology (e.g., when a call went off, the responsible staff was informed via a personal pager versus wireless phone). Each hospital has somewhat different mechanisms and focuses on monitoring patient safety initiatives (e.g., fall prevention protocols, regular rounding for patient comfort and safety). However, no study has systematically investigated the differences of the effect of adopting different call light-related devices on patient safety outcomes.

In an effort to minimize the length of time to complete the staff surveys, a limited number of questions were included. The reliability and validity information about this survey is limited, including threats to internal validity. Further instrument development is needed to develop items that inquire about the tasks that need to be handled by a nurse versus an aide as the most appropriate helper, and the perceived urgency level of each call light use reason. Also, it could be helpful to use focus groups to solicit nurses' and patients' opinions.

The researcher recognized that additional factors may also be critical to staff's response time to call lights, including the efficiency of teamwork among a patient's responsible staff members, staff's priority among their assigned tasks, patients' acuity levels, and changes in a patient's physical condition and mental status. However, these issues are not addressed in this paper as study limitations. In addition, since patient self-reported outcomes regarding call light usage and responsiveness (e.g. consumer assessment, inpatient satisfaction) could not be used to show its concordance with the results of the nurse self-reported measures used in this study, it is a study limitation. This limitation should be taken into account for future research directions.

Future studies should consider including human resource factors in the analysis of the perceived nature of patient- and family-initiated call lights, including staffing patterns (e.g., total nursing hours per patient day by different shifts) and skill mix (e.g., the registered nurse/unlicensed nursing personnel ratio, the usage rate of sitters). Pressing research focuses include investigating the relationships of staff's perceptions about the nature of call lights with NQF-endorsed^® ^outcome measures (e.g., falls prevalence, falls with injuries), post-discharge patient satisfaction scores (e.g., the Hospital Consumer Assessment of Healthcare Providers and Systems, also known as HCAHPS; the monthly inpatient satisfaction survey questionnaire), and the call light use and responsiveness information recorded from the patient room call light tracking system (e.g., Responder^® ^IV, manufactured by Rauland: http://www.rauland.com/ResponderIV.cfm). In addition, it would be essential to link staff's perceived nature of call lights with patient satisfaction and clinical outcome indicators, and to explore and develop cause-and-effect relationships between these variables, if any. It is also crucial to examine the characteristics of the patients for whom the nurses provide care and its differences across hospitals.

## Conclusions

It was assumed that if answering call lights was more highly prioritized among nursing tasks, a staff member would perceive call lights as being important to patient safety, requiring nursing staff's attention, and being meaningful. If so, the action of answering call lights should not be perceived as preventing staff members from doing the critical aspects of their role. However, this multihospital study found that only 49% of the participants perceived that patient-initiated call lights mattered to patient safety, only 52% thought that these calls required the attention of nursing staff, and up to 53% of the participants thought that answering call lights prevented them from doing the critical aspects of their role.

Obviously, additional efforts are necessary to reach the ideal or even a reasonable level of patient safety-first practice in current hospital environments. To endorse patient-centered care, nursing executives and unit managers must promote the effectiveness of patient-initiated call light use and the efficiency of staff's responsiveness to call lights. Regular on-the-job training of patient safety-first practices with a focus on addressing patients' call lights would be required to raise consensus on and awareness of the perceived important levels of call lights among staff members.

## Competing interests

The author declares that she has no competing interests.

## Authors' contributions

THM for the entire project.

## Pre-publication history

The pre-publication history for this paper can be accessed here:

http://www.biomedcentral.com/1472-6963/10/52/prepub

## Supplementary Material

Additional file 1 Survey Questionnaire. Click here for file
