# Supplementary material for: Perspectives of staff nurses of the reasons for and the nature of patient-initiated call lights: an exploratory survey study in four USA hospitals
Source: BMC Health Serv Res. 2010 Feb 26;10:52. doi: 10.1186/1472-6963-10-52 (PMC2841165; doi:10.1186/1472-6963-10-52)
Supplement: Additional file 1 — Survey Questionnaire. [file 1472-6963-10-52-S1.DOC]

Please answer the following questions.

1. Your gender:

1 Male _0_ Female

2. Your age: __________ years

3. Your highest completed education program:

1 High school or diploma

2 Associate degree

3 Bachelor degree

4 Master’s degree or higher

5 Other, please indicate_____________

4. What type of unit do you currently work in?

1 Acute medical care unit

2 Acute surgical unit

3 Acute medical-surgical unit

9 Other, please indicate ___________________

5. You work as a:

1 Staff nurse

2 Nurse aide, nurse technician, or medical assistant

3 Unit clerk or administrative assistant

4 Advanced Nurse Specialist/Nurse Practitioner

6. Your primary working shift is:

1 Day shift

2 Evening shift

3 Night shift

4 12-hour day shift

5 12-hour night shift

6 Rotating

7 Other, please indicate_____________________

7. How many years have you been working in acute inpatient care units? ______ years

8. How many patient-initiated calls per hour do you alone respond to? About _____ calls

9. How many patient-initiated calls per hour do you and your team members (e.g., nurse, nurse aide, nurse technician, or medical assistant) respond to? About _____ calls

10. On average, how long will it take to answer a patient-initiated call light?

During the day shift: About _________minutes and ________ seconds

During the evening shift: About _________minutes and ________ seconds

During the night shift: About _________minutes and ________ seconds

11. Do most of the call lights require nursing staff’s attention and nursing care?

1 Yes _0_ No

12. Are most of the reasons for call lights meaningful?

1 Yes _0_ No

13. Does answering call lights prevent you from doing the critical aspects of your role?

1 If yes, please tell us why ____________________________________________

0 No

14. Do most of the call lights matter to patients’ safety during hospital stays?

1 Yes _0_ No

15. **The reasons of patient-initiated call lights to you and the prevalence of each situation, by percentage (from 0% to 100%, e.g., 25%; the percentages do not need to add up to 100%):** **Please select all that apply.**

1 Extremely urgent medical problem (e.g., a life-or-death emergency call)

The prevalence of such situations is about ______ % of the calls.

2 Bathroom, bedside commode, or bedpan assistance

The prevalence of such situations is about ______ % of the calls.

3 Intravenous problems or pump alarm

The prevalence of such situations is about ______ % of the calls.

4 Pain medication and management

The prevalence of such situations is about ______ % of the calls.

5 Repositioning, transfer, or mobility assistance

The prevalence of such situations is about ______ % of the calls.

6 Personal assistance (e.g., for food, water, changing clothes or bed sheets, housekeeping)

The prevalence of such situations is about ______ % of the calls.

7 Obtaining information (e.g., patients’ health status, medications, discharge planning)

The prevalence of such situations is about ______ % of the calls.

8 Getting nurses’ attention for no specific reason (e.g., for fun)

The prevalence of such situations is about ______ % of the calls.

9 Demanding a nurse’s presence or a companion at bedside for no specific reason

The prevalence of such situations is about ______ % of the calls.

10 Accidentally pushed the call light (did not mean to call nurses)

The prevalence of such situations is about ______ % of the calls.

11 Other I, please indicate_______________________

The prevalence of such situations is about ______ % of the calls.

12 Other II, please indicate______________________

The prevalence of such situations is about ______ % of the calls.

16. Please share with us the matters or issues that have higher priority than answering patient-initiated call lights. ________________________________________________

**Thank you for your time and participation!**

Please put the completed survey in the included envelope, seal the envelope and drop it into the large envelope located in the nurses’ lounge labeled “The Call light Survey.”
